# Synthesis and adsorption properties of chitosan-silica nanocomposite prepared by sol-gel method

**DOI:** 10.1186/s11671-014-0722-1

**Published:** 2015-02-28

**Authors:** Tetyana M Budnyak, Ievgen V Pylypchuk, Valentin A Tertykh, Elina S Yanovska, Dorota Kolodynska

**Affiliations:** Chuiko Institute of Surface Chemistry of National Academy of Sciences of Ukraine, 17 General Naumov Str., 03164 Kyiv, Ukraine; Faculty of Chemistry, Taras Shevchenko National University of Kyiv, 64 Volodymyrska Str., 01033 Kyiv, Ukraine; Faculty of Chemistry, Maria Curie Skłodowska University, M. Curie Skłodowska Sq. 2, 20-031 Lublin, Poland

**Keywords:** 78.67.Bf, 78.67.Sc, 81.07.Pr, 82.35.Np, 81.16.Be, Nanocomposite, Sol-gel method, Chitosan, Adsorption, Vanadium, Molybdenum, Chromium

## Abstract

A hybrid nanocomposite material has been obtained by *in situ* formation of an inorganic network in the presence of a preformed organic polymer. Chitosan biopolymer and tetraethoxysilane (TEOS), which is the most common silica precursor, were used for the sol-gel reaction. The obtained composite chitosan-silica material has been characterized by physicochemical methods such as differential thermal analyses (DTA); carbon, hydrogen, and nitrogen (CHN) elemental analysis; nitrogen adsorption/desorption isotherms, scanning electron microscopy (SEM); and Fourier transform infrared (FTIR) spectroscopy to determine possible interactions between silica and chitosan macromolecules. Adsorption of microquantities of V(V), Mo(VI), and Cr(VI) oxoanions from the aqueous solutions by the obtained composite has been studied in comparison with the chitosan beads, previously crosslinked with glutaraldehyde. The adsorption capacity and kinetic sorption characteristics of the composite material were estimated.

## Background

Nowadays, there is a great interest in the development of low-cost sorbents for water remediation. The use of low-cost sorbents has become an alternative to expensive methods such as membrane filtration or ion exchange [1,2]. Recently, numerous approaches have been studied for the development of cheaper and more effective adsorbents containing polysaccharides [3,4].

Natural polysaccharide chitosan, a derivative of chitin [5-11], is of great interest as an organic component in the composites developed for water treatment because of the high quantity of amino and hydroxyl groups, which is very important for sorption processes [12-19]. Moreover, chitosan possesses such properties as good biocompatibility, high adhesion to the surface, a wide range of pH stability, and expressed chelating properties. It has been proved that chitosan is an effective bioadsorbent towards some toxic ions, dyes, and organic contaminants [20-23]. Silicas are characterized by advanced surface stability in the acidic medium and highly developed surface, acceptable kinetics, thermal stability, resistance to microbial attack, and low cost.

Various methods of preparation of hybrid materials based on inorganic materials and polysaccharides such as chitin and chitosan for different applications have been studied [24-30]. The method of obtaining chitin and chitosan hybrid mesoporous composites applied for dye removal from natural waters and industrial effluents was presented in [31]. The authors of [32] described the adsorption of chitosan on the nanosilicas (SiO_2_, TiO_2_/SiO_2_, and Al_2_O_3_/SiO_2_). Preparation of the hybrid chitosan-silica sorbent was carried out in [33], and the obtained nanocomposite was used in high-performance liquid chromatography. The chromatographic results showed that the obtained hybrid chitosan-silica sorbent and silica gel encapsulated by chitosan have similar properties according to the separation efficiency. The authors of [34,35] studied the preparation of zirconia-chitin-based composites and their possible application as a template for the *in vitro* formation of zirconium dioxide nanophase from ammonium zirconium(IV) carbonate under extreme conditions. For that purpose, novel zirconia-chitin-based composites were prepared using hydrothermal synthesis. It was proved that chitin can be effectively silicified by the two-step method with the use of Stöber silica micro- and nanodispersions under extreme biomimetic conditions [36]. In that case, the chitin-silica composites obtained at 120°C and characterized by the presence of spherical SiO_2_ particles homogeneously distributed over the chitin fibers.

Sol-gel reaction has been extensively studied for several decades as a method to prepare ceramic precursors and inorganic glasses at relatively low temperatures. The major advantage of the process is that mild conditions, such as relatively low temperature and pressure, are used in this type of ceramics processing. Within the past years, the sol-gel process was widely used to create novel hybrid nanoscaled materials based on organic and inorganic components [37-40]. For obtaining such composite materials, the sol-gel reaction is carried out in the presence of organic molecules that are typically polymeric, contain functional groups, and could be immobilized on the inorganic component. Simplicity and possibility of variation of the quantitative ratio of reagents, as well as the nature of initial materials, provide a wide range of applications of the materials. One of the possible applications for those organic-mineral composites is sorption processes and treatment of water solutions from toxic substances.

Peculiarities of preparation of chitosan-silica hybrid materials by using a sol-gel process in a wide range of silica content were studied [41], as well as the properties of prepared hybrid materials towards adsorption of dyes [42] and rare earths [43]. Moreover, recent studies in that field have shown that composites of silica and chitosan [44-49] can be used for extraction and concentration of toxic metals from solutions. However, systematic investigations of adsorption properties of chitosan-silica composites, in particular an effect of nature and the pH of solutions as well as determination of achievable values of adsorption capacity, are necessary.

This work describes the synthesis of the nanocomposite material chitosan-silica for their use as a biosorbent. The hybrid material was obtained by the sol-gel method using tetraethoxysilane (TEOS) as the SiO_2_ precursor. Adsorption properties of the obtained hybrid material were studied with respect to highly toxic metals, such as vanadium(V), molybdenum(VI), and chromium(VI) oxoanions, which are common contaminants in industrial waste waters. Conditions connected with the optimum pH value of the medium, interaction time, and adsorption capacity were studied.

## Methods

Chitosan (No 417963, Sigma-Aldrich, St. Louis, MO, USA) with a molecular weight from 190,000 to 370,000 Da, degree of deacetylation not less than 75%, and solubility of 10 mg/ml and 99.9% tetraethoxysilane precursor (Sigma-Aldrich, St. Louis, MO, USA) were used for the synthesis. All chemicals purchased from Sigma Aldrich were reagent grade.

Composite chitosan-silica was obtained by the sol-gel method through hydrolysis of tetraethoxysilane in the chitosan solution. Composite chitosan-silica was synthesized by following technique: 30 ml of ethanol, 1 ml of distilled water, and 0.5 ml of concentrated hydrochloric acid were added to 46.5 ml of tetraethoxysilane. The obtained mixture was stirred using the magnetic stirrer MM-5 for 10 min and slowly added dropwise to the previously prepared chitosan solution (0.5 g of chitosan was dissolved in 100 ml of 2% acetic acid) and stirred for a day. After 7 days, when the sol became mature, the obtained substance was dried at 60°C.

Thus, in the case of the 100% yield of silica generation by the sol-gel reaction from the tetraethoxysilane precursor, the theoretical mass ratio of the obtained organic and mineral components of the composite was as follows: chitosan:silica = 1:25.

Partially crosslinked chitosan beads were obtained applying the following technique: 2.5 g of chitosan was dissolved in 85 ml of 2% acetic acid and the solution was stirred using the magnetic stirrer MM-5 for 2 h and allowed to stand for 2 days. The obtained solution was added dropwise into the concentrated ammonia solution. After that, the obtained chitosan beads were washed with distilled water several times until neutral pH was achieved. The obtained chitosan beads were placed in 12.5 ml of 0.25% solution of glutaraldehyde in water and heated at 50°C for 2 h. Such a quantity of glutaraldehyde is proper for the crosslinking of 5% of accessible amino groups of polymer. The crosslinked chitosan beads were washed with distilled water and dried at 50°C.

Buffer solutions with pH 1.0 prepared from the standard titrimetric substance of HCl acid, pH 2.5 and pH 5.0 from glacial acetic acid, and pH 8.0 were prepared from 17 ml of 1 M acetic acid and 5 ml of 25% ammonia solution, adding distilled water up to 1 l. The pH values of all buffer solutions were controlled by pH-meter.

Fourier transform infrared (FTIR) spectra of the samples of the initial chitosan and reaction products were recorded using an IR spectrometer with Fourier transformation (Thermo Nicolet Nexus FT-IR, Waltham, MA, USA). For this purpose, the samples were ground in an agate mortar and pressed with KBr.

The concentration of chitosan in the composite was determined by the thermogravimetric method on the derivatograph Q-1500 MOM (Mateszalka, Hungary) with the computer data registration in the temperature range of 15°C to 1,000°C. The samples heating rate was 10°/min. The differential thermal analyses (DTA), TG, and DTG curves were recorded simultaneously.

The specific surface area and the average pore diameter of the composite were determined with the ASAP 2405 (Micromeritics Instrument Co., Norcross, GA, USA). The isotherm plots were used to calculate the specific surface area and the average pore diameter of the chitosan-silica composite.

Elemental analysis of the chitosan-silica composite was carried out by using a carbon, hydrogen, and nitrogen (CHN)/O analyzer (Series II CHNS/O Analyzer 2400, PerkinElmer, Waltham, MA, USA). The analysis was carried out at the combustion temperature of 925°C and the reduction temperature of 640°C.

The surface morphology of the chitosan-silica composite was observed by using a scanning electron microscope (SEM, LEO 1430VP, Carl Zeiss, Inc., Oberkochen, Germany).

The investigations of the adsorption properties of the obtained composite with respect to V(V), Mo(VI), and Cr(VI) oxoanions were carried out in static mode with periodic hand stirring. The sample of 0.1 g of synthesized adsorbent contacted with 25 ml of solutions at different concentrations of salts: NH_4_VO_3_, (NH_4_)_6_Mo_7_O_24_ · 4H_2_O, (NH_4_)_2_Cr_2_O_7_ which were prepared according to [50]. Photometric studies of equilibrium solutions were performed according to the methods described in [51] using a SF-46 spectrophotometer (LOMO, St. Petersburg, Russia) with square cuvettes (optical path length *l* = 1 cm).

### Calculations

The degree of adsorption (*R*) was calculated using the formula:

$$ R = \left({m}_{\mathbf{ads}}/{m}_{\mathbf{o}}\right) \cdot p\ 100\ \% = \left({m}_{\mathbf{o}}-m\right)/{m}_{\mathbf{o}} \cdot p\ 100\ \%, $$

where *m*_0_ is the weight of the metal in the initial solution, *m*_ads_ is the weight of the adsorbed metal, and *m* is the weight of metal in the solution after adsorption equilibrium, which was calculated as *m* = *С* · *V*, where *С* is the equilibrium concentration of metal and *V* is the volume of equilibrium solution.

## Results and discussion

### Physicochemical characteristics of the composite

The sol-gel process can be viewed as a two-network forming process, the first step being the hydrolysis of silicon alkoxide and the second consisting in a polycondensation reaction. Most interest in this method is focused on metal-organic alkoxides, especially silica since they can form an oxide network in organic matrices. The sol-gel reactions of alkoxysilane can be described as follows [37]:

If these sol-gel reactions are complete, fully condensed silica is obtained in this process that can be summarized by the following equation:

If these sol-gel reactions are complete, fully condensed silica is obtained in this process that can be summarized by the following equation:

$$ \mathrm{S}\mathrm{i}{\left(\mathrm{OR}\right)}_4 + 2{\mathrm{H}}_2\mathrm{O}\underrightarrow{{}^{\mathrm{H} + \mathrm{or}\ \mathrm{O}\mathrm{H}\hbox{-} }}\kern0.5em {\mathrm{SiO}}_2 + 4\mathrm{R}\mathrm{O}\mathrm{H} $$

There are many different synthetic techniques used in the sol-gel process to generate polymer-silica hybrid materials. One of them is the *in situ* formation of an inorganic network in the presence of a preformed organic polymer. Those hybrid materials possess strong chemical bonds (covalent or ionic) between the organic and inorganic phases. Also, physical- or weak-phase interactions can be observed between phases, for example, hydrogen bonding or van der Waals attraction.

**Figure 1:**
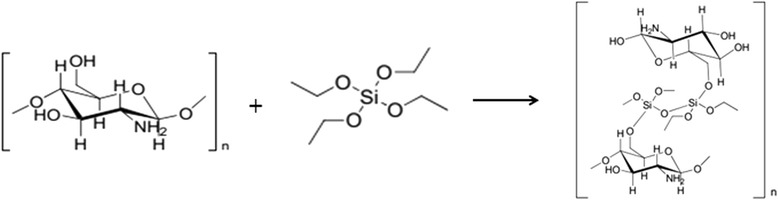
**The scheme of synthesis of chitosan-silica composite.**

The polysaccharides, such as chitosan, are polyhydroxy compounds because they are composed of numerous monosaccharide residues. Their hydroxyl groups could form hydrogen bonds or get into the condensation reaction with silanol groups produced in the course of hydrolysis of the precursor, thus providing silica nucleation on macromolecules [20,37]. The presence of amino groups in the molecule of chitosan facilitates the hydrolysis of TEOS and condensation of created silanol groups, as well as the reaction of silanol groups of silica with carbonyl groups of polymer with creation of Si-O-С bonds. Calculations in the gas phase (B3LYP/6 − 311 + G*) revealed a reaction-free energy (∆*G*_298_) close to 0 kcal/mol for the reactions of alkoxysilanes with hydroxyl groups of cellobiose [52]. Nevertheless, from the related cases, it is known that activation energy is necessary for the initiation of similar reactions [53]. Therefore, the alkoxysilane is not only covalently bound to the chitosan but is also incorporated into the matrix via hydrogen bonding. Although the amine groups do not form covalent bonds with the alkoxysilane under the employed reaction conditions, they act as excellent hydrogen-bonding partners and facilitate the formation of a network and subsequently of a film. Another factor which influences the formation of such a network is pH which is chosen for materials preparation. Under the acidic conditions employed, the amine group of chitosan is protonated and forms an NH_3_^+^ ion which, in turn, is an even better hydrogen-bonding partner than an uncharged NH_2_ group [29,30]. The scheme of synthesis of chitosan-silica composite by the sol-gel method is presented in Figure 1.

**Figure 2:**
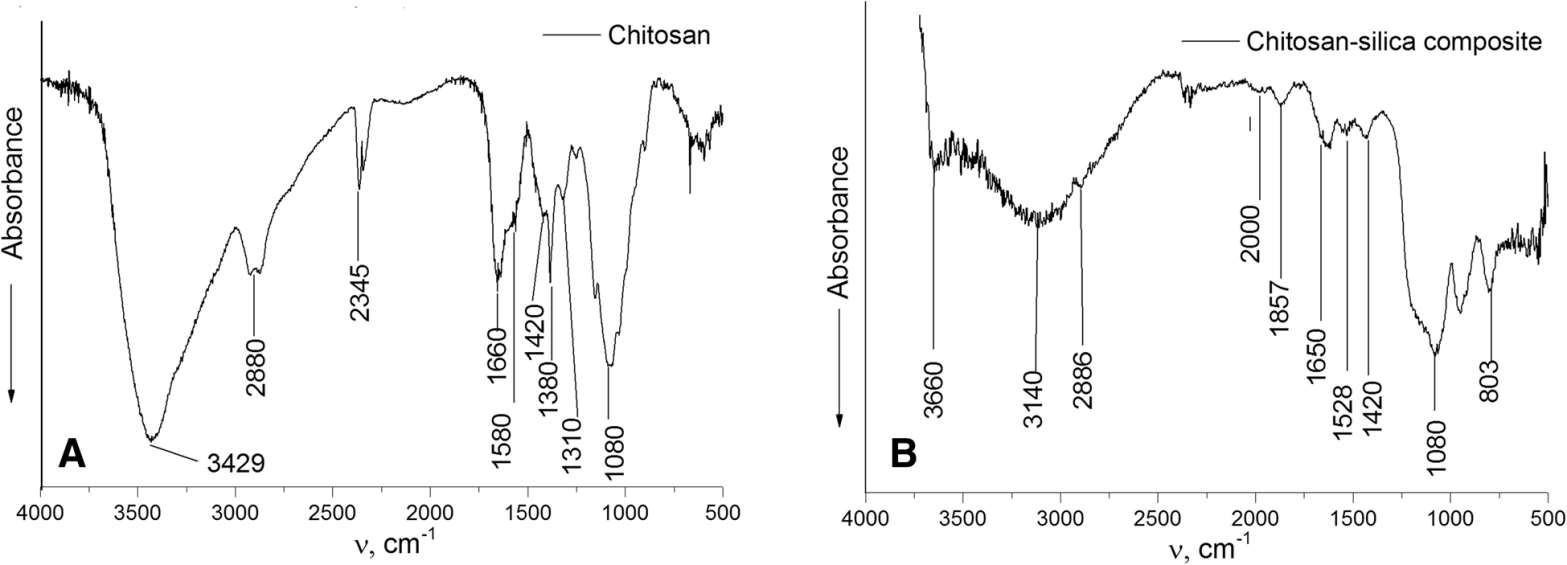
**FTIR spectra of chitosan (A) and chitosan-silica composite (B).**

In order to confirm the SiO_2_ creation as a part of the hybrid composite chitosan-silica, FTIR spectra were obtained for the initial chitosan and synthesized composite (Figure 2). In the FTIR spectrum of chitosan (Figure 2A), the band at 3,429 cm^−1^ corresponds to the stretching vibrations O-H of hydroxyl groups bound with carbon atoms. Intensive absorption bands at 2,800 to 3,000 cm^−1^ are observed due to the С-Н stretching vibrations. The band at 1,580 cm^−1^ corresponds to the deformation vibrations of -NH_2_; 1,420 and 1,380 сm^−1^ for C-H bending vibrations, 1,310 сm^−1^ for asymmetric С-О-С stretching vibrations, and 1,080 сm^−1^ for С-О stretching vibration of СН-ОН were observed.

The FTIR spectrum of the synthesized composite (Figure 2B) has shown a shift of the band 1,528 сm^-1^ of -NH_2_ deformation vibrations in comparison with the spectrum of the initial chitosan. An intensive absorbance at 1,100 сm^−1^ represents the Si-O stretching vibrations.

**Figure 3:**
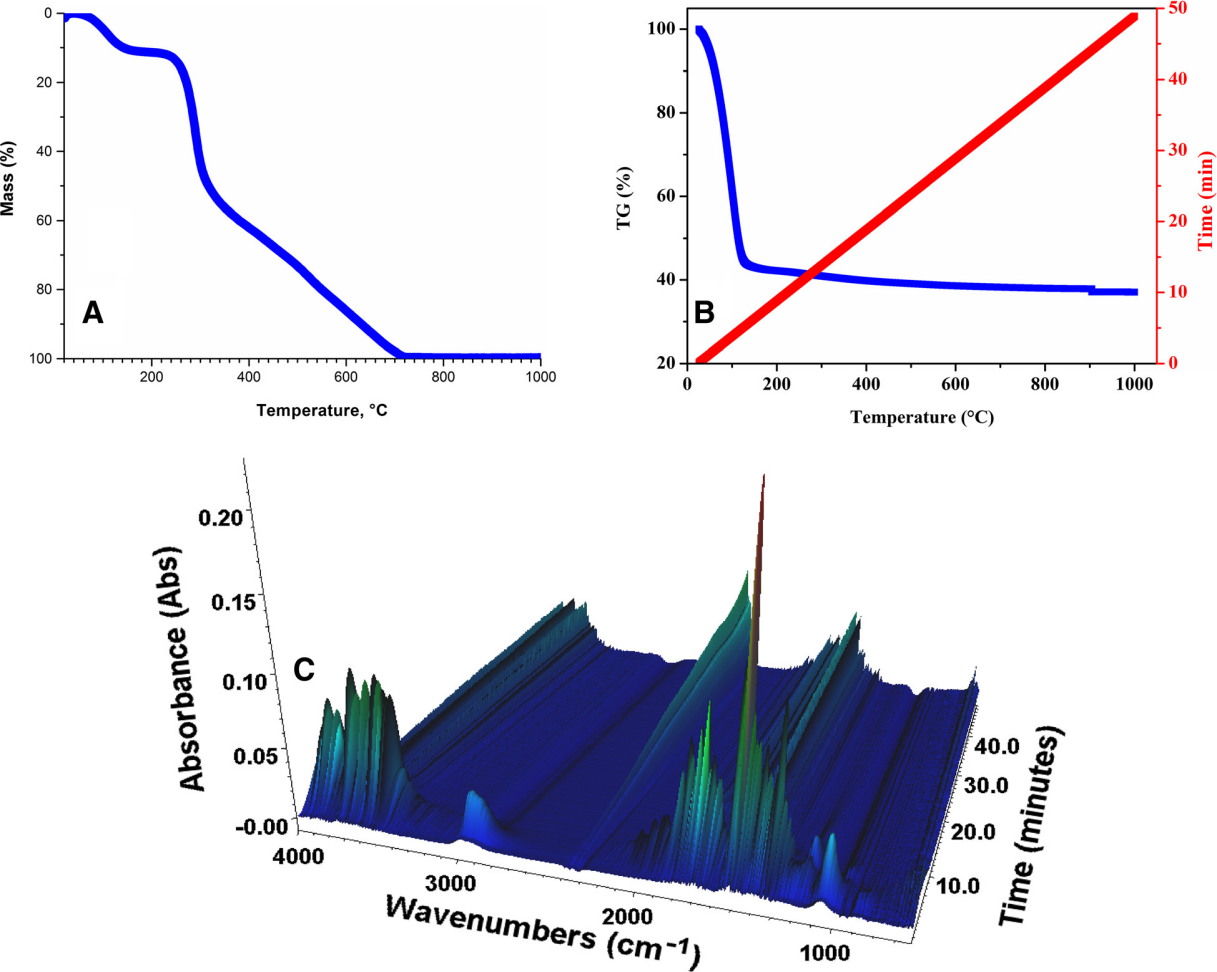
**TG-curves of chitosan (A) and composite (B) and FTIR-TG of chitosan-silica composite (C).**

The FTIR-TG analysis of synthesized organic-inorganic composite was conducted in order to determine the mass ratio of chitosan and silica in the composite (Figure 3). For the TG-curve of chitosan (Figure 3A), two decomposition temperatures can be found. The initial weight loss of 11% from room temperature (30°C) up to 190°C corresponds to the release of adsorbed water. The second recorded decomposition region (190°C to 1,000°C) completely applies to weight loss of chitosan.

The thermogravimetric curve of the chitosan-silica composite (Figure 3B) is characterized by the decomposition region from 30°C to 200°C which is similar to that of the initial chitosan and corresponds to water desorption followed by decomposition of the organic part of the composite. At higher temperatures (200°C to 1,000°C), the process of condensation and elimination of hydroxyl groups could also take place. At that temperature range, decomposition of chitosan was also confirmed by the increasing stretching vibration of C-O in the molecule of CO_2_ at 2,350 cm^−1^ (Figure 3C). Thus, the comparison of TG-curves of chitosan and chitosan-silica composite shows that in the temperature range from 200°C to 1,000°C weight losses of the chitosan-silica composite reach about 10%, which are most likely caused by destruction of the organic component of the composite and hydroxyl groups.

Comparing the results of TGA and mass ratio of initial components for the synthesis, the quantity of chitosan in the composite is in the concentration range from 38 to 100 mg/g.

**Figure 4:**
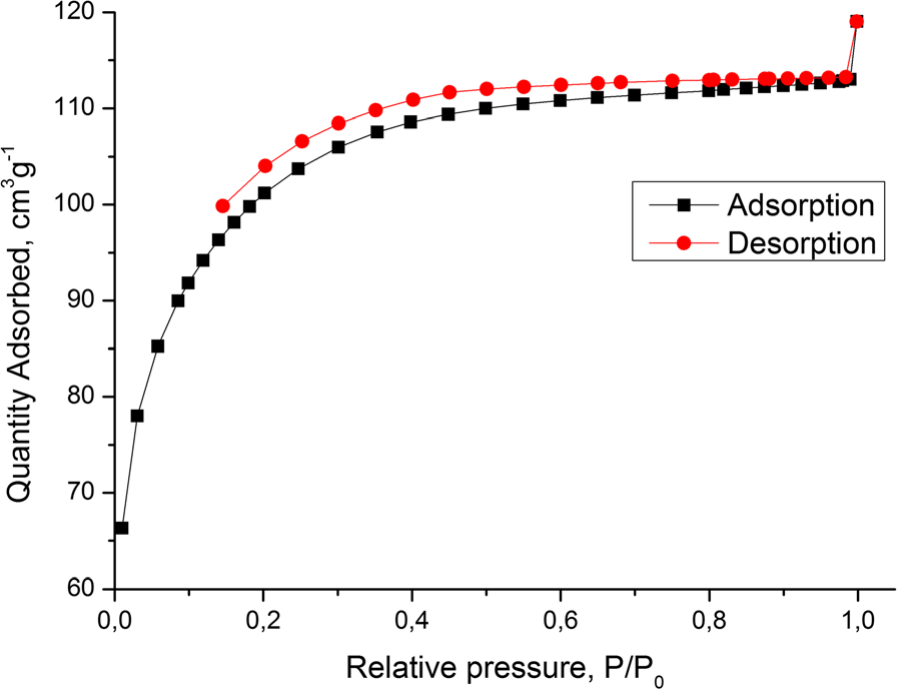
**Nitrogen adsorption/desorption isotherms of the composite chitosan-silica.**

**Figure 5:**
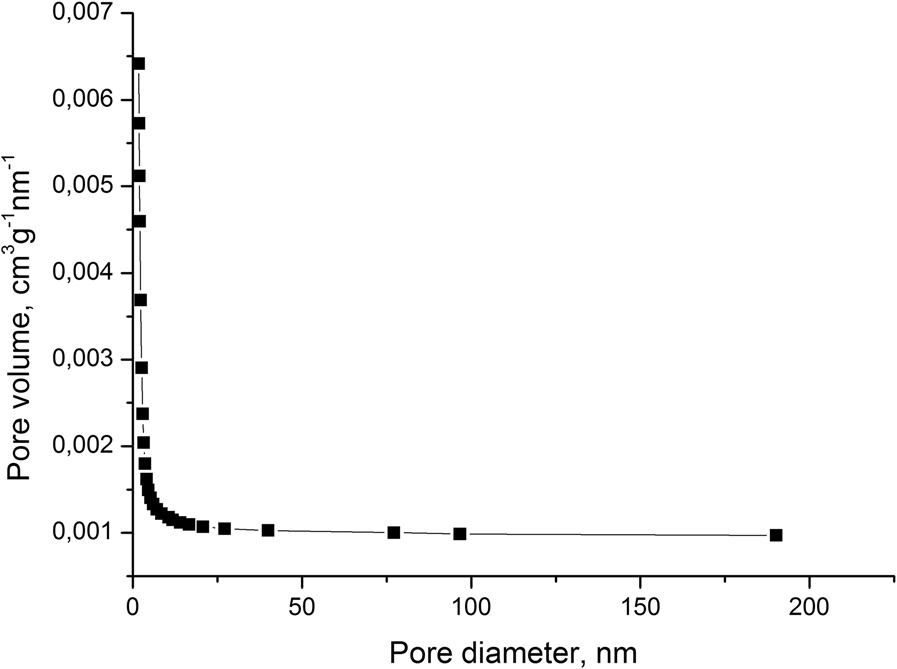
**Pore-size distribution curve for the composite chitosan-silica.**

**Figure 6:**
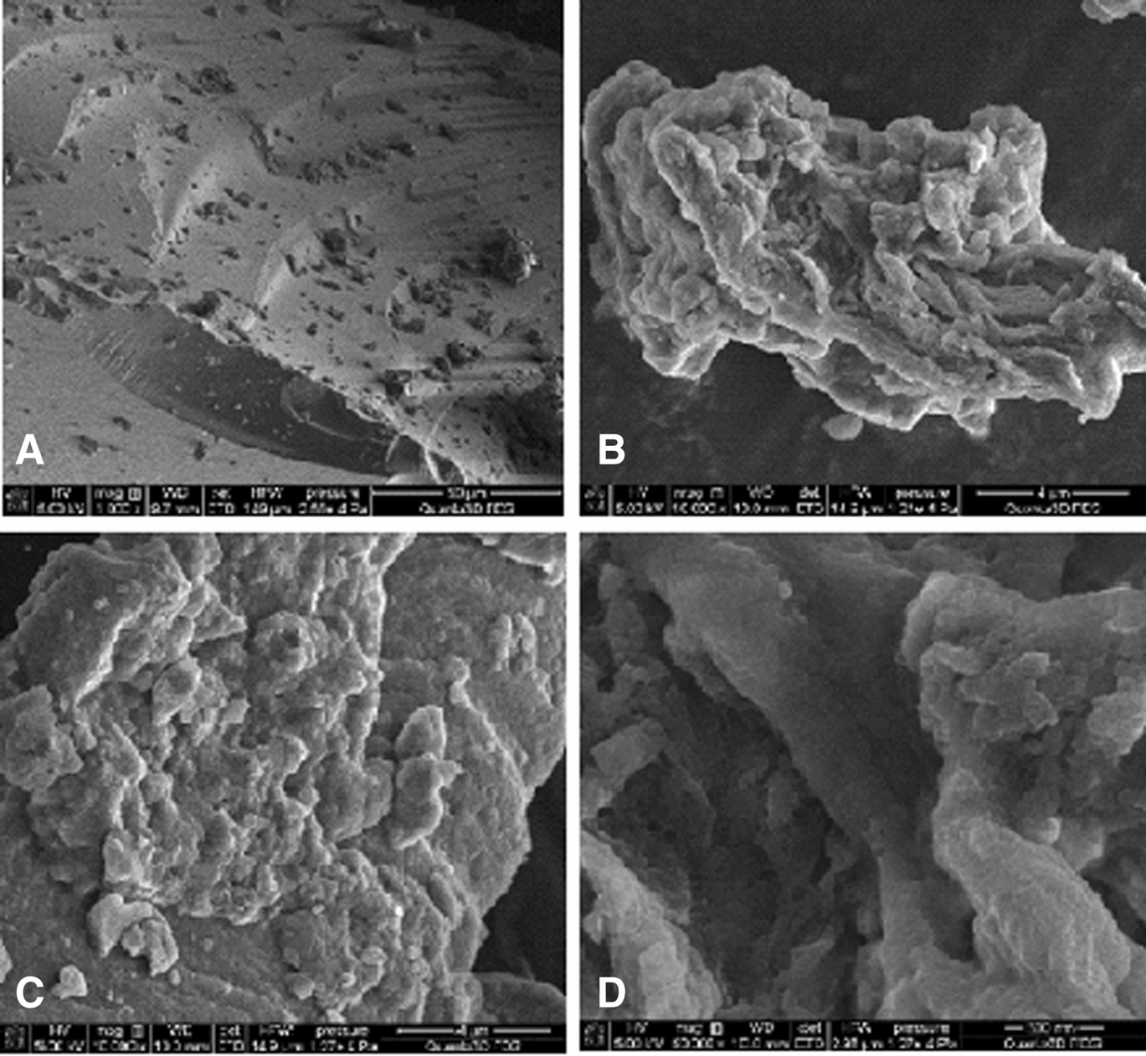
**The SEM images of chitosan-silica at 1,000 (A), 10,000 (B,C), and 50,000 (D) magnifications.**

Figure 4 presents the nitrogen adsorption/desorption isotherms measured at 77 K for the composite chitosan-silica. The shape of the isotherm corresponds to the Langmuir isotherm, type I of the International Union of Pure and Applied Chemistry (IUPAC) classification. This type of isotherm is commonly observed in microporous materials, of which the steep increase of adsorbed quantity at low relative pressure indicates that the available microporous volume is occupied. The presence of micropores is confirmed by the diagram of pore size distribution (Figure 5), which was obtained by the adsorption branch of the isotherm using the BJH method. According to the IUPAC recommendations, the micropores are defined as pores with a diameter not exceeding 2 nm; mesopores are pores with a diameter between 2 and 50 nm, and macropores represent pores with a diameter larger than 50 nm [52]. According to the results of surface area and average pore diameter analyses, the chitosan-silica composite has the BET surface area 359 m^2^/g and the average pore diameter 2 nm (Figure 5). The SEM images showed that the chitosan-silica composite (Figure 6) has a rough and irregular surface.

Carbon, hydrogen, and nitrogen elemental analysis results for chitosan and chitosan-silica are summarized in Table 1. The organic-inorganic composite showed a significant increase in the C/N ratio compared to chitosan. This confirms that the obtained chitosan-silica composite contained an unreacted amount of TEOS precursor.

**Table 1:** **Percentage of carbon, hydrogen, and nitrogen and the C/N ratio for the chitosan-silica composite**

**Adsorbent**	**C (%)**	**H (%)**	**N (%)**	**C/N ratio**	**Reference**
Chitosan	40.72	7.25	7.33	5.55	[5]
Chitosan-silica composite	8.71	8.00	0.38	22.92	This article

### Influence of pH on adsorption

**Figure 7:**
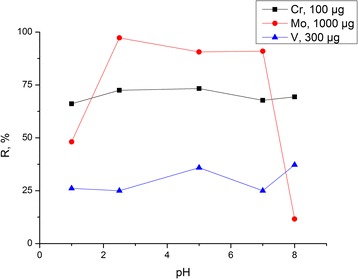
**Degree of adsorption of V(V), Mo(VI), and Cr(VI) oxoanions by composite chitosan-silica as a function of medium acidity.**

Investigation of sorption properties of the synthesized composite began with determination of medium acidity for the highest removal of the studied ions. The degree of adsorption of V(V), Mo(VI), and Cr(VI) oxoanions by composite chitosan-silica as a function of the medium acidity is presented in Figure 7. It can been seen that the highest degree of adsorption (97.25%) on the surface of the obtained composite was observed for Mo(VI) oxoanions from the solution containing 1 mg of molybdenum in the acidic medium (pH 2.5, acetic acid). At the same initial concentration of the metal in the solution, 90.6% to 90.95% of Mo(VI) oxoanions were concentrated by the chitosan-silica composite from acidic (pH 5.0, acetic acid) and neutral media. A decreasing extent of adsorption of hexavalent molybdenum was observed in the case of adsorption from the strong acetic medium formed by hydrochloric acid (pH 1.0) and from a slightly alkaline solution (pH 8.0) formed by ammonium acetate buffer corresponding to 48.12% and 11.61%, respectively. Chromium(VI) oxoanions were extracted on the surface of the obtained composite with an approximately equal degree of adsorption, but the highest adsorption was in the pH range from 2.5 to 5.0, formed by acetic acid in the case of the initial concentration of metal solution 4 μg/cm^3^. The lowest values of adsorption degree (25% to 37%) were found for vanadium(V) oxoanions at the initial concentration of metal solution 12 μg/cm^3^ (Table 2).

**Table 2:** **Degree of adsorption of V(V), Mo(VI), and Cr(VI) oxoanions by composite chitosan-silica as a function of medium acidity**

**Oxoanion**	**Degree of adsorption (%)**				
	**pH 1.0**	**pH 2.5**	**pH 5.0**	**Distilled water**	**pH 8.0**
	**HCl**	**СН**_**3**_ **СООН**	**СН**_**3**_ **СООН**		**Ammonium acetate buffer**
V(V)	26.09	25.00	35.87	25.00	37.23
Mo(VI)	48.12	97.25	90.60	90.95	11.61
Cr(VI)	66.07	72.48	73.30	67.75	69.37

Experimental conditions: mass of sorbent—0.1 g, volume of solution—25 ml, weight of vanadium in the initial solution—0.3 mg, weight of molybdenum in the initial solution—1 mg, and weight of chromium in the initial solution—0.1 mg.

Thus, the synthesized composite showed adsorption activity with respect to the investigated ions in different pH ranges, but the highest degree of adsorption was observed in the acidic medium. The values of medium acidity, at which the maximum adsorption activities of the chitosan-silica composite for each of the studied oxoanions were achieved, correspond to the published data of complexation conditions of these ions with amino groups of chitosan in solutions [21].

### Influence of initial metal ion concentration on adsorption

**Figure 8:**
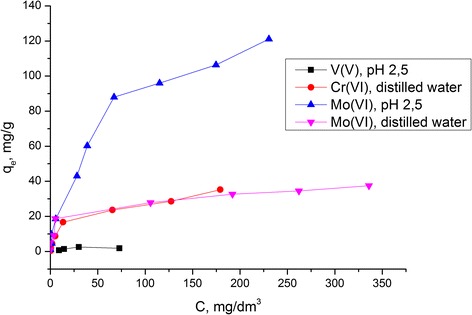
**Adsorption isotherms of V(V), Mo(VI), and Cr(VI) oxoanions on the chitosan-silica composite.**

Adsorption isotherms in the static mode for each ion were obtained for calculation of the values of the adsorption capacity of the composite (Figure 8). The chitosan-silica composite quantitatively extracted 100 and 500 μg of molybdenum from the acidic and neutral media, respectively. By increasing the initial mass of molybdenum, the degree of sorption of molybdenum(VI) was decreased in the neutral (from 90% to 34%) and acidic (from 93% to 60%) media. In the acidic medium (pH 2.5, acetic acid), the composite removes the mixture of ions MoO_4_^2−^, [Mo_6_O_21_]^6−^, and [Mo_7_O_24_]^6−^ with the maximum adsorption capacity 121.0 mg/g (or 1.26 mmol/g) and 37.4 mg/g (or 0.39 mmol/g) of molybdenum ions MoO_4_^2−^ from neutral solutions.

From the distilled water, the chitosan-silica composite removed НCrO_4_^−^ ions in the amount of 35.2 mg/g (0.68 mmol/g). But the composite adsorbed these ions with the highest degree of adsorption (94%) at the initial solution concentration 8 μg/cm^3^.

The synthesized chitosan-silica composite showed the lowest adsorption activity with respect to vanadium(V) oxoanions at pH 2.5, which were presented as a mixture of ions [V_10_О_28_]^6−^, [НV_10_О_28_]^5−^, and [Н_2_V_10_О_28_]^4−^; the adsorption capacity in that case was 2.5 mg/g (or 0.05 mmol/g). The chitosan-silica composite quantitatively adsorbed VO_3_^−^ ions in the neutral medium in the case of metal concentration up to 20 mg/dm^3^, but at the higher concentration (until 80 μg/cm^3^), V(V) oxoanions were extracted at 90%. Adsorption isotherms of all investigated ions belong to the *L*-type, where the ratio between the concentration of the compound remaining in the solution and the concentration adsorbed on the solid decreases when the solute concentration increases, providing a concave curve suggesting a progressive saturation of the solid [22]. *L*-isotherms of the Langmuir model are common for the monolayer adsorption, where the adsorbed layer is one-molecule thick.

**Figure 9:**
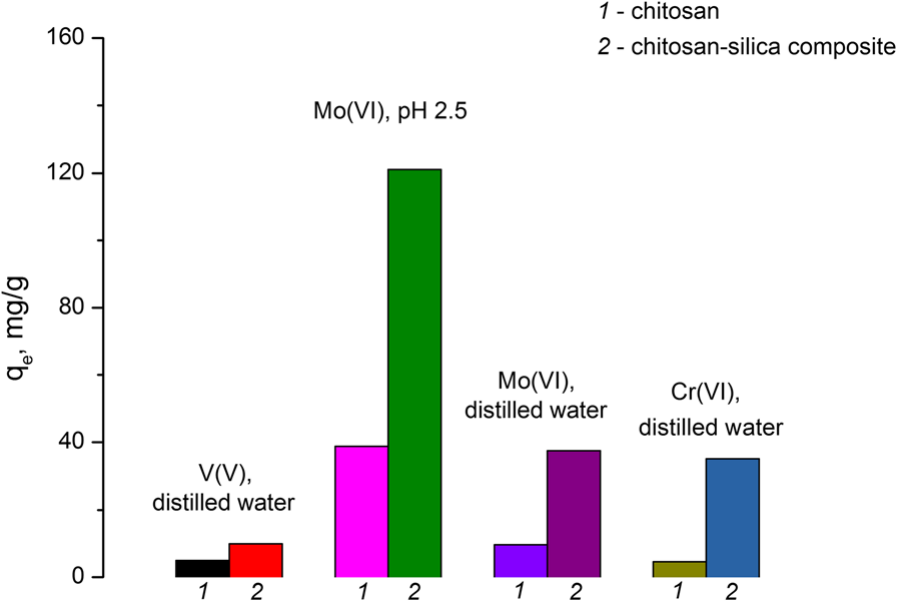
**Comparison of adsorption capacity of chitosan (** ***1*** **) and composite (** ***2*** **).** Comparisons were calculated per 1 g of chitosan with respect to studied oxoanions.

The obtained values of the adsorption capacities of the chitosan-silica composite and the partially crosslinked chitosan beads with respect to V(V), Mo(VI), and Cr(VI) oxoanions from solutions with different concentrations of metals are shown in Table 3. The data indicate that chitosan has a higher adsorption capacity compared to that of the chitosan-silica composite. In spite of this, taking into account that the obtained composite includes chitosan from 38 to 100 mg/g, the contribution of organic part to the maximum adsorption capacity (*q*_*e*_) with respect to each of the studied ions was estimated (Figure 9). A *q*_*e*_ of 1 g of initial chitosan with respect to V(V) oxoanions from the neutral medium is 5 mg/g, but under the same conditions, a *q*_*e*_ of 1 g of immobilized chitosan is 10 mg/g, which is almost twice higher than the adsorption capacity of the chitosan beads.

**Table 3:** **Adsorption capacity of chitosan-silica composite compared to a partly crosslinked chitosan**

**Metal**	**pH**	**Ionic form**	**Chitosan-silica composite**		**Partially crosslinked chitosan beads**	
			**mmol/g**	**mg/g**	**mmol/g**	**mg/g**
Vanadium	2.5	[V_10_О_28_]^6−^, [НV_10_О_28_]^5−^, [Н_2_V_10_О_28_]^4−^	0.05	2.5	3.59	183.1
Molybdenum	2.5	MoO_4_^2−^, [Mo_6_O_21_]^6−^, [Mo_7_O_24_]^6−^	1.26	121.0	4.04	387.9
	Distilled water	МоО_4_^2−^	0.39	37.4	1.0	96.5
Chromium	Distilled water	НCrO_4_^−^	0.68	35.2	0.95	49.4

Experimental conditions: mass of sorbents—0.1 g and volume of solution—25 ml.

In the acidic medium generated by acetic ions (pH 2.5), partially crosslinked chitosan adsorbs molybdenum with *q*_*e*_ 39 mg/g, but under the same conditions, the immobilized chitosan extracts three times more than chitosan beads (*q*_*e*_ is equal to 121 mg/g). It is interesting that in the neutral medium 1 g of partially crosslinked chitosan concentrated four times less molybdenum than the 1 g of chitosan that was part of the chitosan-silica composite: 10 and 37 mg/g, respectively. The synthesized composite adsorbs chromium(VI) in the neutral medium with an adsorption capacity of 35 mg/g which is seven times higher than that of a partially crosslinked chitosan of 5 mg/g.

An increase in adsorption capacity of immobilized chitosan compared to partially crosslinked chitosan could be explained by the expanded quantity of accessible adsorption sites of the chitosan-silica composite and high surface area, as well as a more suitable morphology of synthesized composite for adsorption of V(V), Mo(VI), and Cr(VI) oxoanions.

### Influence of contact time on adsorption

**Figure 10:**
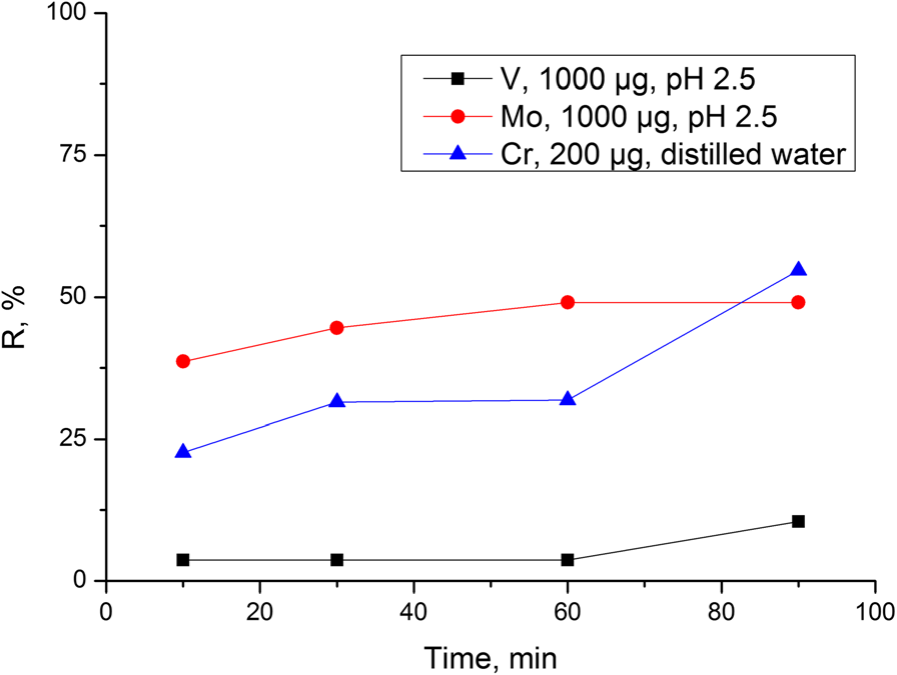
**Dependence of adsorption (** ***R*** **,%) on time of contact with the solutions.** Dependence of adsorption (*R*,%) on time of contact with the solutions containing 1 mg of molybdenum at pH 2.5, 1 mg of vanadium, and 200 μg of chromium in the neutral medium.

According to the obtained results for all studied ions (Figure 10), the degree of adsorption consistently increases for several hours, but the maximum degree of adsorption of all studied ions by the composite surface is achieved in a day which is typical of polymeric adsorbents where the sorption characteristics are defined by interactions between the ions and the functional groups of supported chitosan.

## Conclusions

The chitosan-silica composite was synthesized by the sol-gel method through the hydrolysis of tetraethoxysilane in the chitosan solution. IR spectroscopy confirmed the fact of Si-O-Si polymeric network formation in the presence of chitosan polymer. According to the thermogravimetric analysis and theoretical calculations of the reaction mixture, the obtained composite contains from 3.7% to 9.1% of chitosan.

The comparison of adsorption capacity of biopolymer chitosan (partially crosslinked with glutaraldehyde) and the synthesis via the sol-gel reaction chitosan-silica composite showed that 1 g of immobilized chitosan has better adsorption capacity than the initial chitosan with respect to the studied oxoanions. The obtained organic-inorganic composite contained a small part of chitosan, in comparison with the inorganic part of silica—1 g of composite included from 38 to 100 mg of chitosan. It was found that a small quantity of polymer in the composition of the composite makes it easier to develop increased adsorption properties and kinetic characteristics towards the studied ions. For instance, adsorption capacity of the synthesized composite increased several times in comparison to that polymer with respect to the following: vanadium—9.9 mg/g in the neutral medium, molybdenum—121.0 and 37.4 mg/g at рН 2.5 and in the neutral medium, respectively, and chromium—35.2 mg/g in the neutral medium. It was shown that the synthesized composite extracted the studied metal ions in a day.
